# Developing Single Nucleotide Polymorphisms for Identification of Cod Products by RAD-Seq

**DOI:** 10.3390/ani10030423

**Published:** 2020-03-03

**Authors:** Shoujia Jiang, Xingyu Ma, Tao Li, Changqing Zhu, Xinxin You

**Affiliations:** 1 BGI Zhenjiang Detection Co., LTD, Zhenjiang 212132, China; jiangshoujia@genomics.cn (S.J.); maxingyu@chinamarine.com (X.M.); litao@chinamarine.com (T.L.); 2 Shenzhen Key Lab of Marine Genomics, Guangdong Provincial Key Lab of Molecular Breeding in Marine Economic Animals, BGI Academy of Marine Sciences, BGI Marine, BGI, Shenzhen 518083, China; 3 Childhood Food Institute, School of Food Science, Nanjing Xiaozhuang University, Nanjing 211171, China

**Keywords:** cod products, RAD-Seq, SNPs, species identification

## Abstract

**Simple Summary:**

As high-value fishery products, cod species are frequently faked by non-cod ones. Genetic fingerprinting is important for both certifying authenticity and traceability of fish species. In this study, we developed a method that combines DNA barcoding and restriction-site associated DNA sequencing (RAD-Seq) approach for the identification of cod products. Two sequences that contain single nucleotide polymorphism (SNP)s were identified and these SNPs can be used to distinguish three different cod species, which are Atlantic cod (*Gadus morhua*), Greenland turbot (*Reinhardtius hippoglossoides*), and Patagonian toothfish (*Dissostichus eleginoides*). This SNP-based method will help us to identify the products, which are sold under the name of “Xue Yu” (Cod) in China, and works in parallel with existing fish identification techniques to establish an efficient framework to detect and prevent fraud at all points of the cod commercialization.

**Abstract:**

The increase in the rate of seafood fraud, particularly in the expensive fishes, forces us to verify the identity of marine products. Meanwhile, the definition of cod lacks consistency at the international level, as few standards and effective application methods are capable of accurately detecting cod species. Genetic fingerprinting is important for both certifying authenticity and traceability of fish species. In this study, we developed a method that combines DNA barcoding and the restriction-site associated DNA sequencing (RAD-Seq) approach for the identification of cod products. We first obtained 6941 high-quality single nucleotide polymorphism (SNP)s from 65.6 gigabases (Gb) of RAD-Seq raw data, and two sequences that contain SNPs were finally used to successfully identify three different cod product species, which are Atlantic cod (*Gadus morhua*), Greenland turbot (*Reinhardtius hippoglossoides*), and Patagonian toothfish (*Dissostichus eleginoides*). This SNP-based method will help us to identify the products, which are sold under the name of “Xue Yu” (Cod) in China, and works in parallel with existing fish identification techniques to establish an efficient framework to detect and prevent fraud at all points of the seafood supply chain.

## 1. Introduction

Fish consumption has been increasing mainly due to an increase in the attention of consumers towards nutrition and health. However, this trend also leads to more fraudulent practices, which chiefly include mislabeling, the substitution of fish with inexpensive or inedible varieties, and fish products of unknown origin [1,2]. To ensure the authenticity of food, most countries have established a legal management system to control the seafood supply chain. The European Union (EU) is one of the global leaders in food traceability. It has developed several legal systems such as requiring the scientific name, corresponding commercial name, production process, source of origin, and fishing gear category to be reported on seafood (EU regulation 1379/2013) [3].

In the past three decades, the number of seafood consumption in China has tripled [4], and high-value alien marine species have become increasingly popular, followed by emerging food safety problems [5]. In Europe, among seafood products sold in Italy and the United Kingdom, the level of seafood substitution varies from low (3.4%) to medium (10.4%). However, the misbranding rate of Belgian restaurants is 54.5%, mainly because other low-priced varieties have replaced high-cost species [6]. In the USA, a large-scale survey involving more than 1200 seafood samples from 674 retailers had found that one-third of the samples were mislabeled. Another study in Los Angeles also found a high percentage (47%) of restaurants had mislabeled [7]. In China, a survey of cod products sold in the Chinese market found that the rate of mislabeling was very high, which exceeded 60% even with the less stringent definition. In that survey, only 42.3% of the samples were *Gadiformes*, while the others were *Perciformes*, *Pleuronectiformes*, or toxic *Tetraodontiformes* species [8].

To solve this big problem, the Chinese government has formulated a series of laws, regulations, and standards for food production and labeling, such as “General rules for the labeling of prepackaged foods” (GB 7718-2011), “General standard for the labeling of prepackaged foods” (GB 7718-2004), “Standards for animal aquatic products” (GB 10136-2015), and “National food safety standard for fresh and frozen animal aquatic products” (GB 2733-2015) [9]. Despite some progress, the fishery sector still suffers from significant legislative and managerial deficiencies. In particular, there is still a lack of specific regulations and technical supports for the labeling of aquatic products and the official reference list of seafood commodity names.

The use of genetic markers as identification tools is receiving increasing attention from biologists [10]. Although morphological [2] and biochemical markers [11,12] are considered valuable, they are unsuited to the identification of neither fish samples cut into pieces (steak and fillet) nor cooked fish products since proteins are heat-sensitive [13]. To date, most molecular markers analysis used for fish identification are based on the amplification of conserved mitochondrial DNA (mtDNA) regions [14]. The most widely used mtDNA genes in product identification studies are cytochrome c oxidase I (COI) [15,16], cytochrome b (cytB) [17], 12S rRNA, and 16S rRNA [18]. Additionally, restriction fragment length polymorphism (RFLP) [19], single-strand conformational polymorphism (SSCP) [20], and random amplified polymorphic DNA (RAPD) [21] were also used for fish species identification. 

The decreasing cost of next-generation sequencing (NGS) makes single nucleotide polymorphism (SNP) a promising molecular marker in animal genetics, genomics, and aquaculture [22]. This opens the door to the rapid generation of genetic marker data sets, whether through the generation and application of SNP arrays or genotyping directly through genotyping by sequencing (GBS) techniques [23,24,25]. Recent advancements in these techniques are contributing significantly to the certification of the fish products, such as common carp (*Cyprinus carpio*) [22], blue catfish (*Ictalurus furcatus*) [26], chinook salmon (*Oncorhynchus tshawytscha*) [27], and Atlantic cod (*Gadus morhua*) [28]. Also, restriction-site associated DNA sequencing (RAD-Seq) is considered as one of the popular methods to develop thousands of SNP loci in non-model organisms with limited genomic resources [29]. The RAD-Seq has been prominently used for the identification of threespine stickleback [30], Atlantic salmon, and sea trout [31]. However, an SNP-based identification by RAD-Seq has not been applied to the recognition of cod to date.

This study developed unique SNP markers based on the RAD-Seq and COI for the detection of three commercial cod species named as “Xue Yu” in the Chinese supermarket. The established detection method was subsequently applied to the actual detection of seafood offered in large-scale stores and the target species were successfully distinguished, which proves that this method could be used to support the standardization of the cod market and the protection of consumer rights and interests.

## 2. Materials and Methods

### 2.1. Cod Samples and DNA Isolation

The cod samples were purchased from local supermarkets, Shenzhen, China. All samples were sent to the laboratory by ice-chilled. Approximately 50 mg tissues of each sample were collected and stored at −20 °C before the use. In order to retrieve and check the data easily, each sample was marked with a unique number and product details, such as sample ID, species on the label, and product status (in Table 1). DNA was extracted using the DNeasy Tissue and Blood Kit (Qiagen Inc., Valencia, CA, USA), followed by the manufacturer’ s recommended guidelines. The quantification of the extracted DNA was also ascertained by weighing the absorbance at 260 and 280 nm in a UV-Vis spectrophotometer [32]. 

### 2.2. Species Identification by COI

For the species identification, all tested samples used for RAD-Seq were firstly amplified by PCR with COI universal primers (F: CACGACGTTGTAAAACGACTCAACYAATCAYAAAGATATYGGCAC; R: GGATAACAATTTCACACAGGACTTCYGGGTGRCCRAARAATCA), sequenced on an automatic sequencer ABI3730 (Thermo Fisher Scientific, Waltham, MA, USA), and then the sequences were aligned using BLASTn tool (https://blast.ncbi.nlm.nih.gov/). PCR for COI amplification consisted of 5 µL 10× PCR Buffer, 0.5 µL of 1–20 ng/µL genomic DNA, 4 µL of dNTPs, 1 μL of a 0 µM solution of each primer1, 0.5 μL of Taq polymerase and 38 μL of ddH_2_O, to a final reaction volume of 50 µL. PCR amplification included the following profile: 95 °C denaturation for 3 min, followed by 35 cycles of 95 °C for 15 s, 55 °C for 15 s, 72 °C for 1 min, and final extension step at 72 °C for 5 min. PCR products were checked by 1.5% agarose gel (Gelly PhorLE, Euro Clone, UK) (120 V, 200 Ma, 25 min). Then, the PCR products were sent to BGI-Guangzhou for purification and sequencing. The cod COI sequences were analyzed using the basic local alignment search tool through the GenBank database (https://www.ncbi.nlm.nih.gov/genbank/) considering sequence similarity of at least 98%. Four mitogenomes of cod species (DQ385443.1 for *Theragra chalcogramma*; JF952739.1 for *G. macrocephalus*; LC146707.1 for *G. ogac*; and JQ353979.1 for *Anoplopoma fimbria*) were downloaded from NCBI (National Center for Biotechnology Information), and then, the COI regions were aligned with 28 COI sequences produced here for the tested samples. We used MEGA7 [33] for the tree construction, using the Bayesian inference analysis. All experiments were carried out according to the guidelines of the Animal Ethics Committee and were approved by the Institutional Review Board on Bioethics and Biosafety of BGI (No. FT14015).

### 2.3. RAD-Seq and Data Analysis

We prepared standard RAD-Seq libraries with 28 samples following the protocol [34]. The 1 µg of genomic DNA from per sample was digested with the restriction endonuclease Taql (5′TCGA3′) incubated for 20 min in Fast Digest buffer (total volume of 20 μL) at 64 °C. Then, a 5 μL RAD adapter mixture (64 barcodes) was added to the interruption product and the ligation homogenization purification. Quantitative analysis was then carried out by the operating instructions of the Qubit detection kit (Invitrogen, Carlsbad, MA, USA). An Agilent 2100 Bioanalyzer (Palo Alto, CA, USA) was used to detect the length distribution of the purified PCR products. Subsequently, the products were cyclized, digested by enzyme digestion, and purified. The purified product was quantified by the Qubit dsDNA HS Assay kit (Thermo Fisher Scientific, Aalst, Belgium). Finally, sequencing was carried out at the BGISEQ-500RS (BGI, Shenzhen, China) using a 100-bp paired-end strategy.

By using the SOAPnuke software (https://github.com/BGI-flexlab/SOAPnuke) [35], all RAD-seq data of each sample were quality constrained. The remaining high-quality reads were used for subsequent analyses. Then, the genotyping analyses were conducted by the non-reference genomes software Stacks v2.4 (http://catchenlab.life.illinois.edu/stacks/) [25]. The Ustacks program (-m 2 -M 2 -p 15) was run to cluster RAD tags until they exactly matched. The Cstacks program (-b 1 -n 3 -p 15) was set to collect variations, and retained reads were sorted into loci by the Sstacks program (-p 15 -b 1). The parameters of retaining SNPs were as follows: (1) base quality ≥ 25; (2) depth ≥ 5. A total of 6941 SNPs were available for the downstream identification analysis. Then, the following criterion was used to obtain the specific SNPs: (1) in the same species, the genotype of each sample was homozygous and more than 80% of samples had the same genotype; (2) the same locus of three cod had different genotypes.

### 2.4. Primer Design and SNPs Validation

To validate the SNPs identified from RAD-Seq, seven of them were selected for PCR amplification. Flanking sequences of selected SNPs were extracted from the *G. morhua* reference genome [36] by the BLAST tool (-p blast -m 8 -e 1e-5) (https://nihlibrary.ors.nih.gov/bioinfo/blast/blast.htm) and PCR primers were designed using the software Primer3 (http://primer3.ut.ee/) [37]. Finally, once primer pairs were selected, we checked their specificity in the Primer-BLAST tool available on the NCBI website (https://www.ncbi.nlm.nih.gov/tools/primer-blast/).

SNP validation was performed using randomly selected fish products, including *Epinephelus coioides* (orange-spotted grouper), *Boleophthalmus pectinirostris* (blue-spotted mudskipper), *G. morhua*, *R. hippoglossoides*, and *Merlangius* from Shenzhen’s supermarkets, and then we used the samples for the subsequent analyses. Genomic DNA was extracted using DNeasy Blood & Tissue Kit (QIAGEN, Dusseldorf, Germany) following the manufacturer’s recommended guidelines [38]. DNA extraction negative control was processed in parallel to ensure sample integrity throughout the DNA extraction procedure. Standard PCRs (conditions: 95 °C denaturation for 3 min, followed by 35 cycles of 95 °C for 15 s, 60 °C for 15 s, 72 °C for 1 min, and final extension step at 72 °C for 5 min) were performed to amplify the selected loci. PCR control reactions were run using ultrapure water. Negative control was performed on *E. coioides*, *B. pectinirostris*, respectively. Validation required SNPs firstly to amplify predominantly and secondly to be polymorphic. Then, the PCR products were sent to BGI-Guangzhou for sequencing and the sequences were aligned using MEGA7 for checking the genotypes.

## 3. Results

### 3.1. DNA Barcoding of Cod Samples 

**Figure 1:**
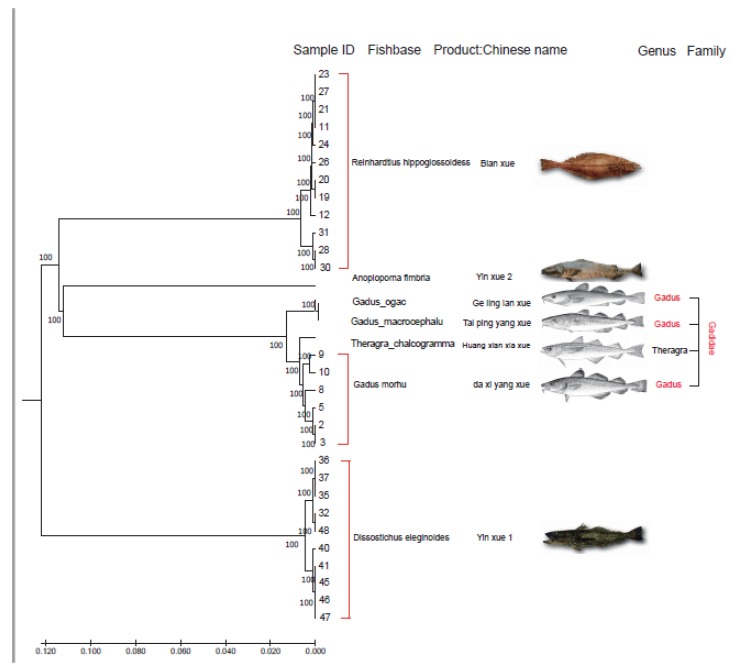
Bayesian tree using COI sequences obtained from common fish species sold as cod in a Chinese supermarket. The fish images were retrieved from Food and Agriculture Organization of the United Nations (FAO) and the codfish name was obtained from Fishbase.

The primary analyses for species identification using 28 COI sequences and the four available mitogenomes showed which samples were suitable to be used for subsequent analyses as standard cod samples (Table 1). The Bayesian tree (Figure 1) clustered 12 samples as Blan xue, ten as Yin xue, and eight samples as Da xi yang xue. Simultaneously, the COI identification results were also validated by the phylogenetic tree. Based on these results, the 28 samples were used for the RAD-seq essays.

### 3.2. SNPs Identification Based on RAD Sequencing Data

A total of 65.6 Gb raw data was generated from 28 samples. After trimming the low-quality raw reads, 41.9 Gb of clean data were retained. Clean reads of separated individuals were deposited in the CNSA (CNGB Nucleotide Sequence Archive) public database (CNP0000707). A total of 6941 high-quality SNPs were used for searching identification markers. After filtering out the SNPs, we found only homozygous genotypes for each sample, and more than 80% of samples had the same genotype. A total of 126 SNPs were selected, and then we chose five tag regions containing different SNP genotypes at the same loci among the three cod species.

### 3.3. Primer Design and SNPs Verification

**Figure 2:**
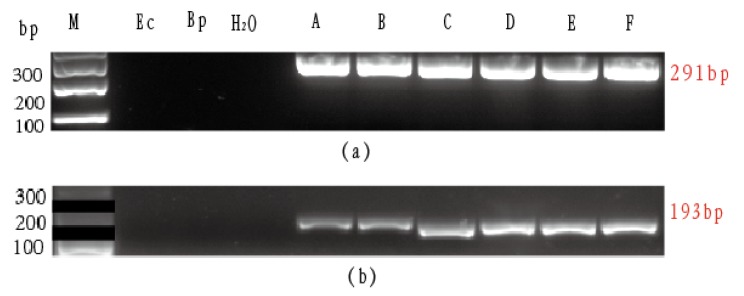
Agarose gel showing the amplification profiles obtained for the two sets of primers tested 12,014 (**a**) and 42,229 (**b**) in the negative controls *E. coioides* (Ec) and *B. pectinirostris* (Bp); in the blank control (H2O); and in *G. morhua*-1 (**A**); *G. morhua*-2 (**B**); *R. hippoglossoides*-1 (**C**); *R. hippoglossoides*-2 (**D**); *D. eleginoide*-1 (**E**); *D. eleginoide*-2 (**F**) samples.

Flanking sequences of those five tags were extracted from the reference genome of *G. morhua* and the existing NCBI data by sequence alignment. Five pairs of primers were obtained under the default stringency conditions of the Primer3 software. Finally, three pairs of primers were failed to yield an amplification product, and two pairs of primers (Table 2) provided effective amplification, which generated amplicons of the expected sizes, without any unspecific amplification bands in the agarose gels (Figure 2). The primer sequences, annealing temperature (TM), and expected sizes for the two tag regions (12,014 and 42,229) are shown in Table 2. We observed no amplification for *E. coioides* (Ec), *B. pectinirostris* (Bp), and blank control (H_2_O), and positive bands for all cod species tested (see Figure 2). The amplicon sequencing and their posterior analyses in the *G. morhua*, *R. hippoglossoides*, and *D. eleginoides* showed differences among the three species. For the 12,014 sequences, we found three SNPs at sites 82, 112, and 220; for the 42,229 sequences, we found two SNPs at 127 and 163 positions (Table 3, Supplementary Material Table S1). In addition, we searched for voucher sequences that matched the sequence F12014 and F42229 from the public databases (Supplementary Material Table S1). We obtained 8 sequences for *G. morhua*, 10 for *D. eleginoides*, and 10 *R. hippoglossoides*, of which the SNPs were consistent with our findings (see Table 3). For the sequence F42229, we obtained eight matches for *G. morhua*, seven for *D. eleginoides*, and four for *R. hippoglossoides*. All SNPs were also consistent with those shown in Table 3.

## 4. Discussion

### 4.1. The Confusion of Commercial Cod Species

Increasing demand for seafood has led to more and more commercial fraud. Fish are often renamed with titles that are perceived to be more attractive, or given the same names as higher-priced species in order to gain economic advantages [39]. An example of this is the English term “Silver cod”, which does not refer to any cod species; the corresponding Chinese Pinyin (Yin Xue) refers to *D. eleginoides*. However, when correctly identified it can be found that the correct English name for this species is Patagonian toothfish, and the corresponding Chinese Pinyin is called “Nan Ji xiao lin quan ya yu”. Similarly, it can be found that the fish known as flat cod does not actually refer to any cod species either, but the corresponding Chinese Pinyin “Ge ling lan bian xue” is referred to *R. hippoglossoides*. In fact, when correctly identified, the English name for the fish known as “flat cod” is Greenland turbot, and the corresponding Chinese Pinyin is “Ma she die” [40]. The Chinese denominations of common codfish samples in the Chinese market with the corresponding pinyin, English and scientific names were provided in Supplementary Material Table S2.

### 4.2. COI Gene and Phylogenetic Analysis

In this study, after careful selection of 28 samples to ensure they met all the requirements of this experiment, it was found that the majority of the sample fragments of the COI gene in the expected size were amplified. A popular method of identifying many fish species is to search for certain similarities in the sequence of databases found in the COI regions [41]. Nevertheless, it is also known that a valuable contribution to clarifying the identity of species in doubtful cases can also be made using the phylogenetic analysis to evaluate the COI gene power of discrimination. Thus, it was used for this study, with a phylogenetic analysis based on the Bayesian inference analysis of 32 sequenced samples for the COI gene and the resulting tree is presented in Figure 1. It can be seen that the tree is divided into three main groups that are a perfect match for the three species. Even though the species *G. chalcogrammus* demonstrates a higher similarity to *G. morhu*, the groups are still well separated. This is in accordance with the discriminant power attributed to COI as the optimal barcode to the identification of animal species [42]. In addition, it has been found that nearly all of these cod species tend to be very similar in appearance, making their morphological identification extremely difficult, and almost to the point of being impossible [22]. Therefore, sequencing all the samples in this study will not just assess the current level of species substitution in this particular type of product, but also more accurately determine species types that can then be used as standard samples in developing subsequent SNP-based methods.

### 4.3. RAD-seq Identification of Cod Species 

When it comes to identifying fish species and even fishery products, molecular methods have been widely used [2], and they have mainly employed mtDNA sequences as taxon barcodes [28,43,44]. Especially, the COI sequence has been serving as a DNA barcode of a global bioidentification system for animals [45]. There are many advantages of the COI barcode: it is less expensive, faster, and only a small segment of mtDNA is needed. Plus, it does not require taxonomic experts to administer. However, the shortcomings of COI barcode are also notable, such as, it cannot distinguish between closely related species from highly similar species; and it cannot help solve the “classification barrier” for the vast majority of unidentified organisms because database resources are lacking [46]. With the rapidly expanding and application of sequencing technology, numerous genomic resources have been provided to identify major marine species [23]. Alternatively, RAD-Seq and subsequent variations have also been extensively applied to generate SNPs [29]. These RAD techniques are popular and accurate, since they are not dependent on prior genomic information [47,48]. Furthermore, they can develop a number of SNPs as candidate markers for species-level assignments [29].

As a final remark, and in what concerns the ecological impacts of our work, our study revealed the use of threatened species as food items. The species *G. morhua* in this study is listed as “near threatened” and its population is susceptible to overfishing [49]. *R. hippoglossoides* is a slow-growing and deep-water fish of high commercial value, which belongs to the family Pleuronectidae [50], yet is now included in the red seafood list of Greenpeace International due to overexploitation [51]. *D. eleginoides* is a large notothenioid species that is considered to have a high migratory capacity [52]. Because of its large size and nutritional content, it is considered to be a kind of fish with high economic value. Most of these species are caught and collected in the distant oceans of Antarctica, which tends to make tracing it very difficult. The above reason will cause a serious existent crisis for the three cod species. Over and above, while there are many detrimental effects for the commercial market of mislabeling seafood, consumers can also be put at risk if they purchase the potentially harmful products. These tools are hence available for the China scientific and governmental authorities to enhance market surveillance and authentication of fresh cod and processed cod products commonly consumed in the country.

## 5. Conclusions

China has the potential to greatly impact the world’s seafood trade and consumption. The current research on cod, chosen as the most commercialized species globally, shows that the lack of national technical support for fishery products could seriously affect the fishing industry, consumers’ protection, as well as the preservation of fish stocks. In this study, the target species have been first detected in identifying cod species with SNPs genotyped by RAD-Seq, which predominantly demonstrates the applicability of this method to the identification of various fish products types. There are several advantages to the use of the RAD-Seq-based method compared to COI analyses. For example, the species-specific SNPs derived from RAD-Seq could be used for designing specific PCR primers that just amplify the corresponding DNA fragment, then it is possible to distinguish the SNPs by agarose gel electrophoresis.

## Figures and Tables

**Table 1:** The successful identification samples and the sequences blast results.

Selected Products	Label of the Received Products			COI Results	
	Sample ID	Species in Label	Product Status	Matched Accession Number	ID GenBank (% Similarity)
Da xi yang xue	NO2	Iceland Atlantic cod	In bulk	LS999407.1	*G. morhua* 99.41%
	NO3	Iceland Atlantic cod	In bulk	LS999407.1	*G. morhua* 99.56%
	NO5	Wild cod	Prepackaged	LS999106.1	*G. morhua* 99.26%
	NO8	Wild cod	Prepackaged	LS999106.1	*G. morhua* 99.56%
	NO9	Norwegian Atlantic cod	Prepackaged	MK011280.1	*G. morhua* 99.55%
	NO10	Norwegian Atlantic cod	Prepackaged	LS999407.1	*G. morhua* 99.41%
Bian xue	NO11	Greenland cod	Prepackaged	AM749133.1	*R. hippoglossoides* 99.12%
	NO12	Greenland cod	Prepackaged	AM749133.1	*R. hippoglossoides* 99.12%
	NO19	Greenland cod	Prepackaged	MH032539.1	*R. hippoglossoides* 99.85%
	NO20	Greenland flat cod	In bulk	AM749132.1	*R. hippoglossoides* 98.38%
	NO21	Greenland flat cod	In bulk	MH032539.1	*R. hippoglossoides* 98.96%
	NO23	Greenland flat cod	In bulk	AM749130.1	*R. hippoglossoides* 98.96%
	NO24	Greenland flat cod	In bulk	HM421730.1	*R. hippoglossoides* 100%
	NO26	Greenland halibut	Prepackaged	KC015874.1	*R. hippoglossoides* 99.85%
	NO27	Greenland halibut	Prepackaged	KF386352.1	*R. hippoglossoides* 100%
	NO28	Greenland halibut	Prepackaged	MH032539.1	*R. hippoglossoides* 98.96%
	NO30	Greenland halibut	Prepackaged	KF386350.1	*R. hippoglossoides* 98.96%
	NO31	Greenland halibut	Prepackaged	MH032539.1	*R. hippoglossoides* 98.96%
Yin Xue	NO32	French cod	In bulk	AB723627.1	*D. eleginoides* 98.97%
	NO35	French cod	In bulk	JN640625.1	*D. eleginoides* 99.54%
	NO36	French toothfish	In bulk	AB723627.1	*D. eleginoides* 98.97%
	NO37	French toothfish	Prepackaged	AB723627.1	*D. eleginoides* 99.56%
	NO40	French toothfish	Prepackaged	AB723627.1	*D. eleginoides* 98.37%
	NO41	French toothfish	Prepackaged	EU752077.1	*D. eleginoides* 98.61%
	NO45	French toothfish	Prepackaged	EF609344.1	*D. eleginoides* 98.47%
	NO46	French toothfish	Prepackaged	AB723627.1	*D. eleginoides* 98.97%
	NO47	French toothfish	Prepackaged	EU074416.1	*D. eleginoides* 98.61%
	NO48	French toothfish	Prepackaged	AB723627.1	*D. eleginoides* 98.97%

**Table 2:** Information of primer for selecting single nucleotide polymorphism (SNP)s.

Primer	Primer Sequence (5′-3′)	Annealing Temperature (°C)	Size (bp)
F12014	CAGATACCCTCGAATA	64	291
R12014	CAAACAAATAGAGGGGTTTGGTA		
F42229	ATTCGGGCAGAACTAAGCCAACCTG	63	193
R42229	CTCATGTTATTTATTCGAGGGAAAGC		

**Table 3:** Two sequences that contain SNPs were chosen for identifying three cod analyzed.

Sequence ID	F12014			F42229	
SNP	82th Base	112th Base	220th Base	127th Base	163th Base
*G. morhua*	G	T	A	G	A
*R. hippoglossoides*	T	A	G	A	C
*D. eleginoides*	A	G	T	T	G

## Supplementary Materials

The following are available online at https://www.mdpi.com/2076-2615/10/3/423/s1, Table S1: The sequence of the specificity SNPs, Table S2: The Chinese denominations of common codfish samples in the Chinese market.
