# Supplementary material for: Developing Single Nucleotide Polymorphisms for Identification of Cod Products by RAD-Seq
Source: Animals (Basel). 2020 Mar 3;10(3):423. doi: 10.3390/ani10030423 (PMC7142540; doi:10.3390/ani10030423)
Supplement: Supplementary file 1 [file animals-10-00423-s001.zip › Table S1. The sequence of the specificity SNPs.docx]

**The sequence of SNP (12014) 1_TCCTCTAATGATCGGTGCACCAGATATAGCTTTCCCTCGAATAAATAACATAAGCTTCTGACTTCTTCCTCCATCTTTCCTGCTCCTTTTAGCATCCTCTGGTGTAGAAGCTGGGGCTGGAACAGGCTGAACTGTCTATCCACCTTTAGCCGGAAACCTCGCTCATGCTGGGGCATCTGTTGATCTCACTATTTTTTCTCTTCATCTAGCAGGGATTTCATCAATTCTTGGGGCAATTAATTTTATTACCACAATTATTAATATGAAACCTCCGGCAATTTCACAGTACCAAACACCCCTATTTGTTTGGAGCAGTACTAATTACAGCT_329 (bp)**

**The sequence of SNP (16364)**

**1_****GTGCACCAGATATAGCTTCCCTCGAATAAATAACATAAGCTTCTGACTTCTTCCTCCATCTTTCCTGCTCCTTTTAGCATCCTCTGGTGTAGAAGCTGGGCTGGAACAGGCTGAACTGTCTATCCACCTTTAGCCGGAAACCTCGCTCATGCTGGGGAGAAAAAATAGTTAGGTCAACGGATGCTCTTC_191 (bp)**

**1_****GTGCACCAGATATAGCTTTCCTCGAATAAATAACATAAGCTTCTGACTTCTTCCTCCATCTTTCCTGCTCCTTTTAGCATCCTCTGGTGTAGAAGCTCGGCTGGAACAGGCTGAACTGTCTATCCACCTTTAGCCGGAAACCTCGCTCATGCTGGGGAGAAAAAATAGTTAGGTCAACGGATGCTCTTC_191 (bp)**

**The sequence of SNP (16364)**

**1_****ATTCGGGCAGAACTAAGCCAACCTGGTGCACTTCTTGGTGATGATCAAATTTATAATGTGATCGTTACAGCGCACGCTTTCGTAATAATTTTCTTTATAGTAATACCACTAATAATTGGAGGCTTTGGGAACTGACTCATTCCTCTAATGATCGGTGCACCAGATATAGCTCATGTTATTTATTCGAGGGAAAGC-193 (bp)**

**The sequence of SNP (1926)**

**1bp_CATCTTCAGGTGTAGAAGCTGGGGTGGAACAGGCTGAACTGTCTATCCACCTTTAGCCGGAAACCTCGCTCATGCTGGGGCATCTGTTGATCTCACTATTTTTTCTCTTCATCTAGCAGGGATTTCATCAATTCTTGGGGCAATTAATTTTATTACCACAATTATTAATATGAGAAATTGCCGGGGGTTTCAT-192bp**
