# Supplementary material for: Developing Single Nucleotide Polymorphisms for Identification of Cod Products by RAD-Seq
Source: Animals (Basel). 2020 Mar 3;10(3):423. doi: 10.3390/ani10030423 (PMC7142540; doi:10.3390/ani10030423)
Supplement: Supplementary file 1 [file animals-10-00423-s001.zip › Table S2. The Chinese denominations of common codfish samples in the Chinese market.docx]

| **鳕鱼Xue Yu Cod** | **Chinese name** | **Chinese pinyin** | **English name** | **FishBase/ASFIS** | **Order** | **Family** | **Genus** | **Mislabeling** | | |
| --- | --- | --- | --- | --- | --- | --- | --- | --- | --- | --- |
|  |  |  |  |  |  |  |  | **Cod=**  **Gadiformes** | **Cod=**  **Gadidae** | **Cod not referable to any specific species** |
|  | **大西洋鳕** | **da xi yang xue** | **Atlantic cod** | ***Gadus morhua*** | **Gadiformes** | ***Gadidae*** | **Gadus** | **√** | **√** |  |
|  | **太平洋鳕鱼** | **Tai ping yang xue** | **Pacific cod** | ***Gadus macrocephalus*** | **Gadiformes** | ***Gadidae*** | **Gadus** |  |  |  |
|  | **格陵兰鳕鱼** | **Ge ling lan xue** | **Greenland cod** | ***Gadus ogac*** | **Gadiformes** | ***Gadidae*** | **Gadus** |  |  |  |
|  | **黄线狭鳕** | **Huang xian xia xue** | **Alaska pollock** | ***Theragra chalcogramma*** | **Gadiformes** | ***Gadidae*** | **Theragra** |  |  |  |
|  | **黑线鳕** | **Hei xian xue** | **Haddock** | ***Melanogrammus aeglefinus*** | **Gadiformes** | ***Gadidae*** | **Melanogrammus** |  |  |  |
|  | **蓝鳕** | **Lan xue** | **Blue whiting** | ***Micromesistius poutassou*** | **Gadiformes** | ***Gadidae*** | **Micromesistius** |  |  |  |
|  | **黄鳕** | **Huang xue** | **Saffron cod** | ***Eleginus gracilis*** | **Gadiformes** | ***Gadidae*** | **Eleginus** |  |  |  |
|  | **江鳕** | **Jiang xue** | **Burbot** | ***Lota lota*** | **Gadiformes** | ***Gadidae*** | **Lota** |  |  |  |
|  | **银无须鳕** | **Yin wu xu xue** | **Silver hake** | ***Merluccius bilinearis*** | **Gadiformes** | ***Merlucciidae*** | **Merlucciinae** |  |  |  |
|  | **北太平洋无须鳕** | **Bei tai pingyang wu xu xue** | **NorthPacific hake** | ***Merluccius productus*** | **Gadiformes** | ***Merlucciidae*** | **Merlucciinae** |  |  |  |
|  | **长尾无须鳕** | **Chang wei wu xu xue** | **Hoki** | ***Macruronus novaezelandiae*** | **Gadiformes** | ***Merlucciidae*** | **Macruronus** |  |  |  |
|  | **细鳞壮鳕** | **Xi lin Zhuang xue** | **Giant grenadier** | ***Albatrossia pectoralis*** | **Gadiformes** | ***Macrouridae*** | **Albatrossia** |  |  |  |
|  | **红鳕** | **Hong xue** | **Red cod** | ***Pseudophycis bachus*** | **Gadiformes** | ***Moridae*** | **Pseudophycis** |  |  |  |
|  | **江鳕** | **Jiang xue** | **Burbot** | ***Lota lota*** | **Gadiformes** | ***Lotidae*** | **Lota** |  |  |  |
|  | **银鳕^1^** | **Yin xue ^1^** | **Patagonian toothfish** | ***Dissostichus eleginoides*** | **Perciformes** | ***Notothenioidei*** | **Dissostichus** |  |  | √ |
|  | **银鳕^2^** | **Yin xue ^2^** | **Black cod** | ***Anoplopoma fimbria*** | **Perciformes** | ***Anoplopomatidae*** | **Anoplopoma** |  |  |  |
|  | **扁鳕** | **Bian xue** | **Flat cod** | ***Reinhardtius hippoglossoides*** | **Pleuronectiformes** | ***Pleuronectidae*** | **Reinhardtius** |  |  |  |
|  | **水鳕** | **Shui xue** | **Oilfish** | ***Ruvettus pretiosus*** | **Scombriformes** | ***Gempylidae*** | **olfische** |  |  |  |
|  | **油鱼** | **You yu** | **Black oilfish** | ***Lepidocybium flavobrunneum*** | **Scombriformes** | ***Gempylidae*** | **Lepidocybium** |  |  |  |

**Table S2.** The Chinese denominations of common codfish samples in the Chinese market. The name was obtained from Fish Base (http://fishdb.sinica.edu.tw/AjaxTree/tree.php), and. Wikipedia.org (www.Wikipedia.org).
